# CIoTVID: Towards an Open IoT-Platform for Infective Pandemic Diseases such as COVID-19

**DOI:** 10.3390/s21020484

**Published:** 2021-01-12

**Authors:** Alfonso P. Ramallo-González, Aurora González-Vidal, Antonio F. Skarmeta

**Affiliations:** Department of Information and Communication Engineering, Faculty of Computer Science, University of Murcia, 30100 Murcia, Spain; alfonsop.ramallo@um.es (A.P.R.-G.); skarmeta@um.es (A.F.S.)

**Keywords:** covid19, IoT platform, speech analysis, spectrogram

## Abstract

The factors affecting the penetration of certain diseases such as COVID-19 in society are still unknown. Internet of Things (IoT) technologies can play a crucial role during the time of crisis and they can provide a more holistic view of the reasons that govern the outbreak of a contagious disease. The understanding of COVID-19 will be enriched by the analysis of data related to the phenomena, and this data can be collected using IoT sensors. In this paper, we show an integrated solution based on IoT technologies that can serve as opportunistic health data acquisition agents for combating the pandemic of COVID-19, named CIoTVID. The platform is composed of four layers—data acquisition, data aggregation, machine intelligence and services, within the solution. To demonstrate its validity, the solution has been tested with a use case based on creating a classifier of medical conditions using real data of voice, performing successfully. The layer of data aggregation is particularly relevant in this kind of solution as the data coming from medical devices has a very different nature to that coming from electronic sensors. Due to the adaptability of the platform to heterogeneous data and volumes of data; individuals, policymakers, and clinics could benefit from it to fight the propagation of the pandemic.

## 1. Introduction

As a result of globalisation, the world becomes interconnected and, in general, the increment of trade and cultural exchange enhances communication, economic growth and stabilises security. As a counterpart, it exposes society to an inter-dependency and it allows infectious diseases to rapidly spread around the world [1,2].

A new coronavirus named Severe Acute Respiratory Syndrome CoronaVirus 2 (SARS-CoV-2) is having a global impact due to its associated disease which is now referred to as COVID-19. On March 11, the global pandemic was declared by the World Health Organization (WHO) (https://www.who.int/director-general/speeches/detail/who-director-general-s-opening-remarks-at-the-media-briefing-on-covid-19—11-march-2020 (accessed on 30 October 2020)). Although there have been other global pandemics in the past, and some others may come in the future [3], some lessons need to be learned. This ongoing pandemic has shown that the appropriate infrastructure, protocols, and treatments for fighting the spread of infectious diseases are still to be developed. This makes it even more important to closely follow up on the stages of the pandemic in order to ensure that the infrastructure is put in place to mitigate the effects of the pandemic in society [4].

One strategy in order to get ahead of the virus involves using digital tools to improve systems that manage real-time data and decision-support systems. The United States Agency for International Development’s (USAID) report *Fighting Ebola with Information*, points out that the lack of standardization and coordination in collecting data from the disease obscured the real picture of the crisis making it inefficient to handle. To prevent a similar situation, a holistic system to identify, gather, analyze data, and use the results to take action, is urgently needed.

In our work, we identify the components of an integrated Internet of Things (IoT) solution that within four layers of an IoT architecture can provide the means to fight pandemics. The result is CIoTVID, a flexible solution that can help policymakers and health care systems to monitor and act over the pandemic. This platform is capable of incorporating different levels of sensorization that is dedicated to manage and analyze the data and to help making decisions in many of the stages of an infectious disease.

Some possible services of our platform are early detection of the disease, monitoring and predicting patients’ evolution, or identifying elderly and at-risk populations. The actions to carry out after this analysis could involve precise lockdown of groups, treating them remotely, or send personalized health communication messages.

The rest of this paper is structured as follows—Section 2 describes the related work and Section 3 delivers material and methods that include the description of each layer of the platform. Section 4 presents the results from the use-case based on analysing human voice for the detection of diseases. Finally, Section 5 concludes the paper.

## 2. Related Work

Our work is based upon the management of data and knowledge extraction related to COVID-19 through the implementation of IoT platforms. The presented use case is based on extracting knowledge related to health conditions from conversations. Consequently, an overview of the literature on COVID-19 data management, IoT platforms and speech recognition is put forward in this section.

### 2.1. COVID-19 Data-Sharing and Management Systems

There exist some global solutions that are proposed to manage data for the coronavirus outbreak. As they are the stepping stone of the platform that is shown in this paper, we provide a description to contextualise the development described in this paper:

The initiative called District Health Information Software 2 (DHIS2), coordinated by the Health Information Systems Programme (HISP), has released an open digital data package for COVID-19 detection, reporting, and surveillance (https://www.dhis2.org/covid-19 (accessed on 30 October 2020)). DHIS2 development brings together a wide variety of data to one central system and it includes indicators, metadata, data quality metrics, national and district dashboards. It is also optimised for mobile use with the DHIS (https://openlmis.org/ (accessed on 30 October 2020)) Android app.

In a different approach, OpenLMIS (https://openlmis.org/ (accessed on 30 October 2020)) is an open-source, cloud-based electronic Logistics Management Information System (LMIS), that is purpose-built to manage health commodity supply chains. The OpenLMIS team has recently focused on supporting national and international COVID-19 response efforts. Countries can plug their supply data so that they have visibility into stock and consumption levels to closely manage limited stocks of commodities.

More related to the protection of institutions considering the cybersecurity, the COVID-19 MISP Information Sharing Community has created a solution for the sharing of information called MISP (https://www.misp-project.org/features.html (accessed on 30 October 2020)). MISP is an open-source threat intelligence platform that due to the COVID-19 pandemic has morphed into a solution for a COVID-19 information-sharing community, focusing on sharing data from Medical information, and Cyber-threats related to COVID-19 in which everyone can contribute and use the data. The possibility of monitoring respiration rates (RR) of COVID-19 patients using WiFi is investigated in Reference [5].

All these initiatives are important as the authors firmly believe that for a solution to be effective against a pandemic has to be open, as there will always be regions that are susceptible of becoming hosts of the virus (and propagators) and may not have the resources to acquire/adopt other solutions with higher economical and technical restrictive overheads.

CIoTVID is human-centered by design and has been given enough flexibility to make possible the integration of all these initiatives previously mentioned. With this, we allow CIoTVID to grow together as the satellite complements grow.

### 2.2. IoT Platforms and Solutions for Monitoring COVID-19

An IoT platform is a multi-layer technology that enables the access and processing of data from different connected IoT devices and it uses the knowledge gained through its analysis to offer a variety of high-level intelligent services [6]. In modern platforms like CIoTVID, this is done taking care of ownership, security, and privacy. The reasons why these platforms give a large marginal value to society is because the proliferation of IoT devices in smart environments is happening in the form of smartphones, smart speakers, security sensors (as part of home alarms), comfort sensors, wearable devices for recognizing and monitoring behavior [7] and so forth. This implies the generation of a variety of methodologies and tools taking the form of integrated IoT platforms in more mature fields such as energy management, home security, or smart entertainment or healthcare.

There already exist some state-of-the-art IoT platforms which are focused on other health conditions different from pandemic diseases. In particular, the Generative Adversarial Network (GAN) based semi-supervised learning approach for clinical decision support in health-IoT platform [8], uses a GAN for improving the quality of labelled data of cerebral strokes. This improves the classification process and, therefore, it facilitates learning about the illness and improving patients’ advice. Also, a standarisation effort is done in Reference [9], where an interoperable Internet of Medical Things (IoMT) platform based on Semantic Web Concepts and the M2M architecture, having doctors as users, is proposed.

A complete review of the architectures, platforms, applications, and industrial IoT-based solutions combating COVID-19 can be found [10]. They highlight three main phases, including early diagnosis, quarantine time, and after recovery. The pandemic has also affected the world of digital technologies, namely IoT, Big Data, Artificial Intelligence and blockchain and it has shaped their development [11]. IoT in particular was used and improved in order to solve the challenge of virus tracing, tracking and spread mitigation. Automatic thermometer scanners in public spaces or tracking apps for positive patients are some examples [12].

Technologies such as CIoTVID, have been used on other fields such as the one regarding energy-focused IoT-platforms. We highlight the one developed by the authors that was given the name of IoTEP [13]. This platform was used in the real world and its outstanding results can be seen in Reference [14]. The work of Terroso et al. shows how the platform made it possible to collect data from a variety of sources, and with the use of complex algorithms provide personalised advice to users. The main addition that we have included concerns about the kind of integrated algorithms and services provided, described in Section 3.3 and Section 3.4. The e-health problems are very different from energy management ones when referred to the intelligence extraction.

IoT platforms have also been dedicated to real-time indoor air quality monitoring [15]. As being COVID-19 a respiratory disease, the indoor environmental conditions of homes will be an important agent for the unraveling of the illness, especially considering that remote clinical assistance seems to be the way forward for several health care systems. Supported by literature that reviews IoT platforms, it was noted that only a few platforms have integrated mechanisms to extract knowledge from data, even in a very simplified fashion [16] and they normally require third parties to perform analytics.

Even though the benefits of executing IoT for fighting COVID-19 pandemic have already been presented [17], to the knowledge of the authors, CIoTVID is the first attempt to create an IoT platform for the management of COVID-19 or any other infectious disease.

### 2.3. Speech Recognition for Diseases

Voice and breathing patterns change while a respiratory disease takes over [18]. The application of machine learning techniques to voice and breathing-related data for the detection of medical conditions is promising and we believe that this knowledge can be leveraged for COVID-19 early detection and development monitoring.

Breathing dynamic patterns, tracked with a low-cost thermal camera, were studied in Reference [19] in order to recognise people’s psychological stress levels. They transformed the uni-dimensional breathing signals into two-dimensional respiration variability spectrogram (RVS) sequences and fed a Convolutional Neural Network in order to create a classifier over some classes created by the k-means algorithm.

Regarding COVID-19 diagnosis, MIT researchers in collaboration with hospitals and biomedical institutions have proposed the use of Artificial Intelligence (AI) transfer learning algorithms trained on cough phone recordings [20]. This follows a similar rationale to our use case and their goal is to test whether such a test can be performed simply through phone-based cough recordings. They are currently collecting data (https://opensigma.mit.edu (accessed on 30 October 2020)) and the used in this preliminary study labeled as COVID-19 is not open. Voice-analysis companies and others are specialising in recognising vocal biomarkers of a variety of diseases, including COVID-19, using machine learning algorithms [21]. Reference [22] is another interesting research work, which summarises the markers in voice recognition systems for detecting COVID-19. Such work focuses more on the proposal of the creation of a mobile application than in the AI algorithms for the recognition of diseases. Finally, Reference [23] collects the efforts related to cough detection, breathing analysis and chat-bots for COVID-19 and other diseases such as asthma and tubercuolosis.

## 3. Materials and Methods

The proposed platform has been structured in four different layers in the digital world in an incremental approach as it has been the common approach for IoT platforms. Substantial development has been done in this new paradigm in the industrial sector. For industry, the paradigm brought by IoT has renamed the environments to Industry 4.0. CIoTVID is an enhancement of RAMI 4.0 (https://www.iso.org/standard/57308.html (accessed on 30 October 2020)) which allows us to use it with the purpose of supervising and helping the development of a pandemic. In the following sub-sections, the description of the platform that allows this new purpose is explained.

### 3.1. Data Collection Layer

The lower part of the CIoTVID is the data collection layer. This layer is in charge of connecting physical devices and actuators to the digital world. Once connected, they will provide data to the platform of the real world (physical world). This will be done via actual sensors or databases that store data from third parties. CIoTVID has been designed taking into consideration the possibility of being used for clinical remote assistance. This required the conception of four different levels of connection with the physical world. The four different levels are as follows:

1. Verbal monitoring with a bot equipped with voice analysis for inference of breathing rhythms, coughing, sneezing, or hissing.
2. The above, plus a dedicated device for oxygen saturation monitoring.
3. The above in addition to hospital equipment as respirators.

It should be noted that the depth levels of monitoring 1 to 2 can be performed at the patient’s home, and provide continuous monitoring of the medical equipment The physical devices that we have considered for our use case can be all connected to the patient’s smartphone. The phone communicates then establishes a secure conduit of communication with CIoTVID which digests the data.

The smartphone is adequate as a hub of any kind of device and serves as the human-platform interface for ensuring that the devices are connected correctly and securely. At the same time, the smartphone can be the support of surveys and questionnaires designed for the patients. These voluntary sources of data are filled by the users or the doctor depending on the service at hand. This will help to add the social dimension to the wealth of data that will be obtained within the IoT ecosystem.

**Figure 1:**
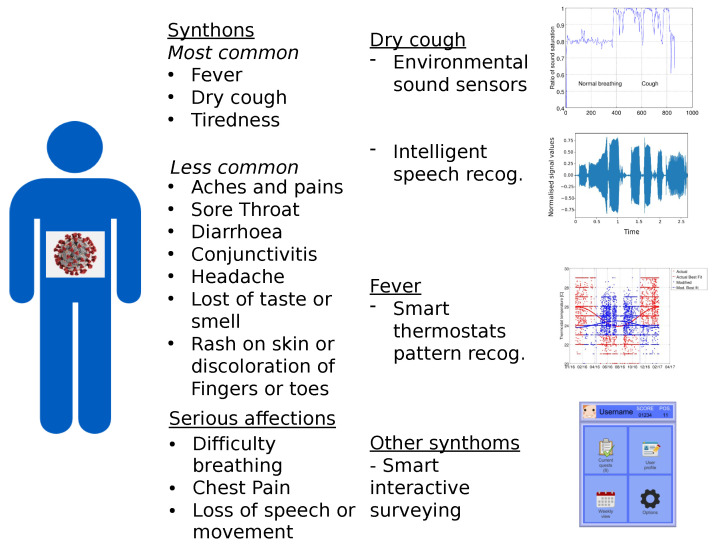
Translation of synthons of COVID-19 into data for the platform. The graph on the top right has been recreated from data of Kunsch et al., 2011 [24]. The rest are from the authors.

**Figure 2:**
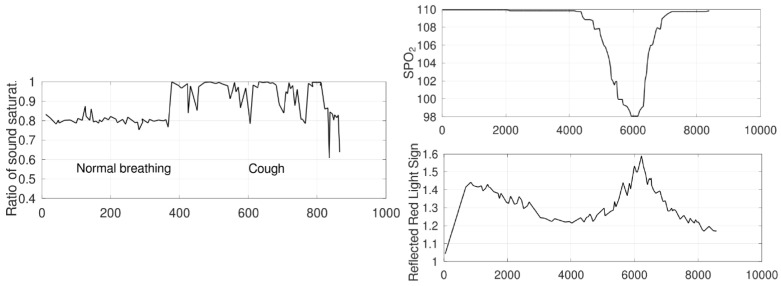
Examples of data coming from Internet of Things (IoT) equipment. **Left**: Sound saturation in an environmental sound sensor when the patient coughs. **Right**: Identification of a drop in Oxygen saturation using a red pulse oximeter. Data from Lee et al., 2016 [25]. The top graph represents the real SpO${}_{2}$ whereas the bottom one represents the readings of the oximeter.

Figure 1 shows the synthons of COVID-19 according to the WHO on mid July 2020 (https://www.who.int/health-topics/coronavirus#tab=tab_3). The synthons allow to envisioned the technologies that could help with the data acquisition of patients. Kunsch et al. [12] showed how the dry cough, symptom more common in COVID-19 can be captured with environmental sound sensors with similar efficiency to on-body sensors. The result in sound saturation in the environmental sensor can be seen in Figure 2, where it is shown how cough relates to other factors and can be detected in different ways. Repetitive coughing could indicate COVID-19 and the environmental sound sensor has a very low cost and could be a passive sentinel for its appearance.

**Figure 3:**
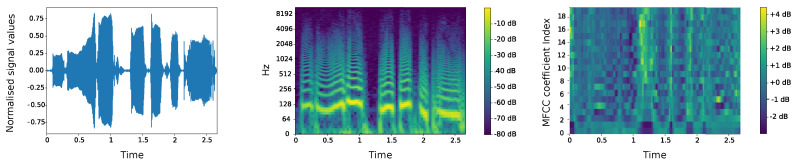
Transformation of a single audio file from its representation in the time domain (with normalised amplitude), to the spectrogram, that is the spectrum of frequencies of the same audio file and finally to the Mel Frequency Cepstral Coefficients (MFCC) coefficient’s representation that is an abstract domain which contains information about the spectral envelope of the speech signal.

Also, the technology of speech analysis in CIoTVID offers the possibility of analyzing these episodes of cough and also the breath rate which could be an indicator of the illness when the cough does not get fully developed. This kind of data transformation can be seen in Figure 3, where the data voice of a patient is normalised (first image). Then, it follows a process based on calculating the spectrum frequencies (second image) and finally creates the MFCC coefficient’s representation (third image). This representation is the input to the algorithm that can, amongst other uses, determine if such an individual is suffering from COVID-19 or not.

**Figure 4:**
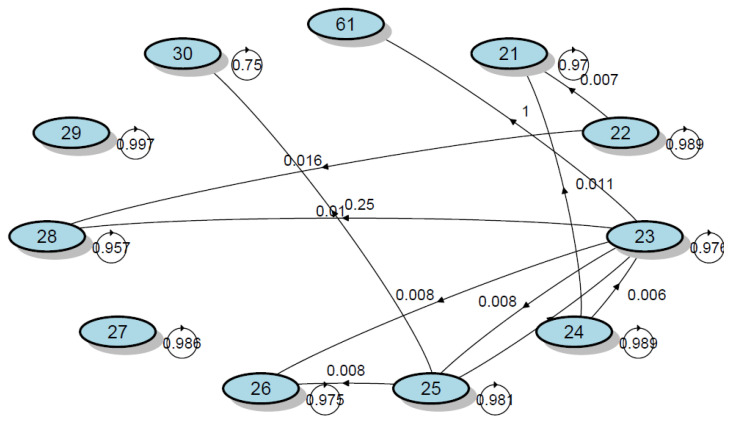
Markov Chain modeling the behavior in the thermostat of a healthy individual. (From unpublished data of the authors).

The next more common synthon is fever. On-body sensors to capture fever are difficult to find and considerably intrusive. The platform offers the possibility of monitoring the set-point temperature of the house to find patterns that could be the result of fever. The authors have developed the first algorithms to model the set-point temperature of the thermostat [26]. These algorithms can be applied with no intrusively to evaluate if the occupants are suffering a change in core temperature. The modeling of the thermostat has been seen work well using Markov Chain on a research work not published at the time of writing. One of the Markov Chains that shows the set point temperature of a Heating Ventilating and Air Conditioning system from a healthy individual can be seen in Figure 4. In that sense, this kind of analysis could be used for behavioural changes in the temperature patterns that can indicate the existence of fever and that sometimes will lead to the diagnose of COVID-19.

When the follow up of patients’ needs to be more close, there exist the possibility of remote assistance using oximeters that can be connected to the mobile phone. These oximeters have been tested thoroughly and are a good way of transforming the physiological parameter of oxygen saturation in the blood into data that can be recognised by a data platform. COVID-19 has a great impact on patients’ capacity of oxygenating. A device capable of capturing this effect is crucial for the follow up of the illness.

### 3.2. Fusion and Storage Layer

Within CIoTVID, all collected data is passed through a stage of homogenization that consists of the transformation of the raw data into NGSI information models (entity attributable models) and JSON. NGSI is a protocol developed by the Open Mobile Alliance (OMA) to manage Context Information. Once transformed, the data will be treated through the FIWARE IoT Agent that supports MQTT and Lightweight M2M protocols. FIWARE is an open solution design for the management of IoT platforms. The FIWARE version of the OMA NGSI interface is a RESTful API via HTTP (https://knowage.readthedocs.io/en/6.1.1/user/NGSI/README/index.html). Its purpose is to exchange context information, mainly in the form of

- One-time queries for context information
- Subscriptions for context information updates

In the case of COVID-19 patients, their voice is stored in wav files that are processed in this layer, so that the MFCC coefficients are stored in the NGSI-based entity-attribute pairs.

The socio-demographic information (gender, age, …), together with the treatment and medicines that the users of the CIoTVID platform are taking is conveniently stored using NGSI models. Users provide a special permission for this kind of sensitive data.

### 3.3. Analytics Support Layer

The third layer of the platform embraces all the functionalities of the platform to provide support for advanced data analytics and services that can run on the upper levels of the platform. This layer is used to host the artificial intelligence and machine learning techniques for analyzing the data. In particular, and with the purpose of evaluating CIoTVID, three features have been proposed in this layer; (1) the computation of a risk factor based on individuals’ context data, (2) the inferring of the development of the illness through anomaly detection in the voice of the users, and (3) the automatic classification of the condition of affected individuals and need of charging at the hospital regarding the level of their conditions.

For this gateway, models have been created for the integration of data from users and IoT personal equipment for health monitoring. This will make possible the utilization of the so-called Digital Therapeutics (DTx), which are scalable, accessible and scientifically validated (Ministerial Statement, Organisation for Economic Co-Operation and Development Health Ministerial Meeting, “The next generation of health reforms”, 2017, http://www.oecd.org/health/ministerial/ministerial-statement-2017.pdf (accessed on 30 October 2020)) for the three objectives mentioned above. In this solution a Smartphone app will initiate traditional clinical supervision and treatment by helping patients manage their condition, including informing when and how much medication to take (WHO global strategy on people-centred and integrated health services, World Health Organisation, 2015). Developments in DTx applied to pandemic episodes such as COVID-19, provide faster diagnosis and better treatments with highly efficient use of resources. Algorithm-based analysis of Big Data and machine learning will provide early detection and diagnosis, treatment, and quality of life (PwC, “What doctor? Why AI and robotics will define New Health”, April 2017. http://www.pwc.com/gx/en/industries/healthcare/publications/ai-robotics-new-health.html (accessed on 30 October 2020)). This work has integrated a machine learning algorithm that is particularly suited to perform these tasks in Big Data environments.

**Figure 5:**
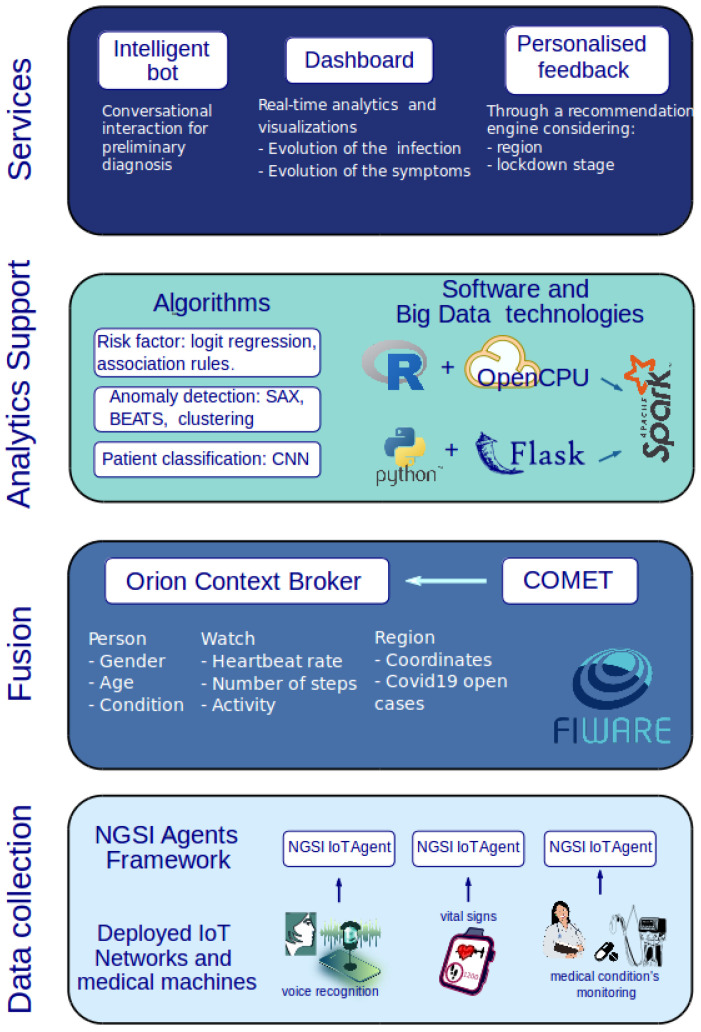
Diagram of the architecture CIoTVID.

The specific IA methods for the three tasks are detailed below:

- Risk factor: To obtain this indicator the algorithms create a logistic regression model that states the risk of a patient suffering severe problems, based on information obtained from data of previous contagious. The method will recognise when they are infected using sociodemographic data and information about other diseases. For this goal, the platform supports the creation of logistic regression models and it has been designed flexible for future more efficient algorithms that may be developed in the future.
- Anomaly detection: In this case, the algorithms will work in a sequential manner, firstly it will extract properties regarding sound files such as audio channels, sample frequency, sample rate, and bit depth and it will normalise the data with respect to those properties. After this, anomaly detection will be performed by two approaches.
  1. Segmentation and representation algorithms such as BEATS [27] that can help in the identification of anomalies.
  2. Representation of the audio file using an advanced spectrogram (non-lineal) in order to visualise the frequency spectrum and identify how it varies in short periods of time. A convolutional neural network can be used in order to extract the characteristics of the images and afterward, some clustering techniques will be available to anomalies. An example of this can be shown in Figure 5.
- Patient classification: The previous analysis could show anomalies related to phenomena that are not related to the individual’s health. In order to discriminate between anomalies, we are going to use the data validated from the questionnaires that state the illness level of the patient. This will help to introduce them as the output of machine learning models for two uses:
  1. Classification of a patient: actual status
  2. Regression of a patient: prediction of the future status.
  The features that are proposed can be enhanced and extended by adding other machine learning algorithms. This layer is generic in the sense that it is conceived to provide analytical support for COVID-19 related issues in many ways, and three of them are described.

CIoTVID could accept the R Project for Statistical Computing, Python, and the unified engine for Big Data processing Apache Spark. They can be chosen depending on the requirements of the analysis and the preferences of the operator. Moreover, off-the-shelf libraries that could be beneficial for the platform could be seamlessly integrated regardless of the language in which they have been created.

The layer described with features shown here has to connect with the higher layer where the business intelligence is created. In CIoTVID this is done based on OpenCPU system for embedded scientific computing which provides a reliable and interoperable HTTP API for data analysis based on R and using Flask, which provides a similar HTTP API for Python. Both of them can be complemented with either sparklyr and PySpark packages respectively when performing distributed Big Data Analytics if required given data volume. The technology provided by these APIs makes it also easy to make available the produced results (output datasets) upon the analysis, which can be of great importance in a pandemic in which several countries are fighting the same enemy around the globe. This will not only provide higher-level information to other users but also, it will make available the data used, and the findings obtained through a set of URLs for other stakeholders to reuse this valuable resource.

Upon the realization of analysis, the produced results (output dataset) are also made available.

### 3.4. Service Layer

This layer serves as an interface between the IoT platform and the different stakeholders that may use CIoTVID. The potential users have been considered during the design of this solution and include policy-makers, managers of health care systems, physicians, infected citizens, and the general public. Depending on the user, the platform will have different services that will be suited-for-purpose, to ensure that each user group receives the right information and provides the right data. Some services that could be provided for the users are:

- An intelligent bot that using conversational interactions recognises synthons to perform a preliminary diagnosis.
- A recommendation engine that provides personalised feedback to users depending on their geographical area and the stage of the lockdown.
- A life report of symptomatology of COVID-19 affected to physicians that allows them to be up to date with respect to the evolution of the synthons of the virus as the virus mutates.
- An evolution of the infection according to the different measures taken to help policymakers evaluate the effectiveness of the actions.
- An economic “what-if” scenarios simulator to provide to stake-holders information about the possible economic consequences of different scenarios to reduce the uncertainties in the most relevant economic sectors.

## 4. Results in Use Case

The performed use case evaluates the validity of the platform CIoTVID in all its stages. To strain the features of the platform, the use case has been done with one of the novel data sources in machine learning, which is the human voice. Those voice recordings are labeled with the medical condition of the speaker. The raw data is introduced in the platform and it will go through a series of treatments until it reaches the AI algorithms that will infer potential conditions in the speaker thanks to the detection of features using neural networks and the final service providing at the business intelligence level of the platform will be the recognition of the health status of a patient.

We have used a subset of recordings extracted from the open ‘Saarbruecken Voice Database’ (Woldert-Jokisz, B. Saarbruecken voice database. http://stimmdb.coli.uni-saarland.de/) composed by the sentence “Guten Morgen, wie geht es Ihnen?” (“Good morning, how are you?”) from 13 healthy people, 14 patients with dysarthrophonia and 13 with laryngitis. Those illnesses were chosen because they share some symptomatology with COVID-19 such as losing voice and difficulties to talk.

In order to incorporate the audios to the platform and fuse them with the rest of the data, we have reproduced them from different mobile devices pretending that they were recorded at the moment. They were then sent to a cloud repository connected to the platform by means of JSON files, which are conformed in the lowest level of the platform. The phone has an encrypted signature that is recognised by the IoT platform, which is in charge of merging the audio file to the metadata of the user. This metadata includes their illness label, gender, age, and others. After that, all the signals are normalised regarding sampling rate and bit-depth values and they are put together in the same dataset in order to proceed to the analysis.

Once the data is fused as described, they are used as inputs to python native algorithms that process the files to produce higher-level information. The novel algorithm to treat voice that has been developed within the project of the creation of this platform has its core in Convolutional Neural Networks (CNN). Given that CNNs are known to work very well with image data, we have transformed the sound into images. The sound is a sequence of vibrations in varying pressure strengths that can be visually represented through a spectrogram, which is the spectrum of frequencies of a signal as it varies with time. The spectrogram of the sound is normally represented with the log scale of the axis because most sounds humans hear are concentrated in very small frequency and amplitude ranges. The advantageous feature of this approach is that spectrograms focus by nature on the repeatability of features making in most cases more visible the existence of irregularities which will be represented in parameters such as breath rate or oxygen saturation levels as arrhythmic signals.

For the purpose at hand, the most popular feature extraction technique in speech recognition is the Mel Frequency Cepstral Coefficients (MFCC) which are derived from a type of cepstral representation of the audio clip (a nonlinear “spectrum-of-a-spectrum”) 2. The steps involved in MFCC are Windowing, computing Fast Fourier Transform (FFT) for each window, generate a Mel Scale, that is separate the entire frequency spectrum into evenly spaced frequencies, and computing Discrete Cosine Transformation (DCT). Finally, the MFCCs are the amplitudes of the resulting spectrum.

For the treatment of the data, each audio file was represented with its MFCC spectrogram, then we divided the dataset into train and test sets and built a CNN. While a CNN can extract its own features, the MFCC features have a long history of success, and providing them to the CNN will greatly reduce the training time while keeping the accuracy high, especially for patterns. We have used a sequential model, consisting of four Conv2D convolution layers, with our final output layer being a dense layer. The output layer had3 which matches the number of labels for our dataset. Our model obtained a training accuracy of 75% and a testing accuracy of 66.67%. Further statistics in order to assess the model are included in Table 1.

A hypothesis test is also computed to evaluate whether the overall accuracy rate is greater than the rate of the largest class. In our case, *p*-value = 0.04 < 0.05 what means that our results are not biased. This is an example of the testing of the platform, but in real operation, the lower part of the platform receives daily or even more frequent voice recordings from each patient what creates the necessity of this tool being implemented on a flexible IoT platform. CIoTVID makes possible the search for indicators of health deterioration and the difference between them over thousands introducing these chains of sound (voice). This use case shows the potential of the platform in order to detect the status of a user that is a suspect of having COVID-19 or also, in order to assess the seriousness or current status of the illness in a patient that has already been diagnosed.

## 5. Conclusions

The pandemic of 2020 due to Cov-SARS-2 has proven that society needs more tools to fight against epidemics. Luckily, technology has evolved far enough to put at the disposition of the health care systems and the governments’ tools based on the digital counterpart of the world. One of the most substantial advantages in this field is the arrival of the Internet of Things (IoT), which brings the opportunity for dedicated and opportunistic sensing. These tools can help provide help to the many with the precision that the traditional methods provided to the few.

The appearance of the Internet of Things (IoT) brings a new paradigm that makes permeable the frontier between the real and the digital world thanks to the existence of more sensors and actuators. As an example, IoT applied to the industry has transformed production. In the case of, health care, professionals and infrastructure can benefit from this technology as it can help on the management of a pandemic thanks to the scalability of ICT solutions. However, to make this benefit a reality and a valuable asset, it is necessary to create tools that connect computer science and the field of digital health.

In this work, we presented the CIoTVID IoT platform. The platform was composed of four layers as the architecture suggested by the RAMI 4.0 convention—one for data acquisition, one for data digestion, one for machine intelligence, and one for services. Each one of the layers was based on state-of-the-art technologies that could provide a complete and systematic workflow from the collection of data and monitoring of patients and resources to the exploitation of data for COVID-19 to provide personal and general services.

To evaluate the validity of the platform, a path from the raw data from the real world to the advice given to the user was followed and evaluated. The result was that the platform was capable of taking the data and to process it in a way that could be used by an AI algorithm. The algorithms that can be hosted in the data analytics layer of the platform performed well, it was capable of performing the check and to identify patients with the same accuracy to clinical tests in which patients need to be fiscally present. It was seen that the platform was capable of delivering information about the results of the AI layer via the services layer, but also it performed well on providing reports about the data processed at the lower levels. The platform based on FIWARE, has its principles and functionalities fully tested from previous works and applications and it showed now that the application of the platform to the issue of a global pandemic could give considerable advantages to policymakers, physicians, and the general public.

Given that CIoTVID is conceived as a multi-purpose platform, it can serve in many ways to the monitoring, detection and ease of Covid-19 impact as specified in the analytics support layer. As a counterpart, the platform is not completely independent since it needs a responsible person that connects and selects the sensors and manages the users in order to start the process, selects the implemented algorithms or implements new ones and so forth. This could make the platform vulnerable, given the sensitivity of the data. The proper mechanisms to avoid the risk of intrusion and lack of privacy have to be taken into account and this is a limitation of the platform.

As for further work, the authors have found that at the time of writing there was no much data available in the form of open data repositories related to the outbreak of COVID-19. It is expected that in the near future there will be a larger number of datasets that could be used on platforms like the one presented here. We believe that valuable further work will be further evaluation of a solution like the one shown here with the new data.

## Figures and Tables

**Table 1:** Sensitivity, specificity, and balanced accuracy per class for the test set.

	Healthy	Dysarthrophonia	Laryngitis
Sensitivity	0.67	1	0.3
Specificity	0.67	1	0.83
Pos. Predictive Value	0.5	1	0.5
Neg. Predictive Value	0.8	1	0.71
Balanced accuracy	0.67	1	0.58

## Data Availability

Data sharing not applicable.
